# Modulation of Dendritic Cells by Microbiota Extracellular Vesicles Influences the Cytokine Profile and Exosome Cargo

**DOI:** 10.3390/nu14020344

**Published:** 2022-01-14

**Authors:** Natalia Diaz-Garrido, Josefa Badia, Laura Baldomà

**Affiliations:** 1 Secció de Bioquímica i Biología Molecular, Departament de Bioquímica i Fisiologia, Facultat de Farmàcia i Ciències de l’Alimentació, Universitat de Barcelona, 08028 Barcelona, Spain; natalia.diaz.garrido@gmail.com (N.D.-G.); josefabadia@ub.edu (J.B.); 2 Institut de Biomedicina de la Universitat de Barcelona (IBUB), 08028 Barcelona, Spain; 3 Institut de Recerca Sant Joan de Déu (IRSJD), 08950 Barcelona, Spain

**Keywords:** bacterial membrane vesicles, exosomes, probiotics, intestinal homeostasis, immune regulation, miRNAs

## Abstract

Gut bacteria release extracellular vesicles (BEVs) as an intercellular communication mechanism that primes the host innate immune system. BEVs from *E. coli* activate dendritic cells (DCs) and subsequent T-cell responses in a strain-specific manner. The specific immunomodulatory effects were, in part, mediated by differential regulation of miRNAs. This study aimed to deepen understanding of the mechanisms of BEVs to drive specific immune responses by analyzing their impact on DC-secreted cytokines and exosomes. DCs were challenged with BEVs from probiotic and commensal *E. coli* strains. The ability of DC-secreted factors to activate T-cell responses was assessed by cytokine quantification in indirect DCs/naïve CD4*+* T-cells co-cultures on Transwell supports. DC-exosomes were characterized in terms of costimulatory molecules and miRNAs cargo. In the absence of direct cellular contacts, DC-secreted factors triggered secretion of effector cytokines by T-cells with the same trend as direct DC/T-cell co-cultures. The main differences between the strains influenced the production of Th1- and Treg-specific cytokines. Exosomes released by BEV-activated DCs were enriched in surface proteins involved in antigen presentation and T-cell activation, but differed in the content of immune-related miRNA, depending on the origin of the BEVs. These differences were consistent with the derived immune responses.

## 1. Introduction

The intestine harbors millions of diverse microbes that, importantly, influence intestinal homeostasis and human health. This microbial community, named the gut microbiota, has crucial functions in the gut and in distal organs [1,2]. The gut microbiome contains more than three million genes that greatly surpass and complement the number of genes in the human genome [3]. In addition to its role in food digestion and energy metabolism, the gut microbiota is crucial for the development and function of the immune system, the integrity of the intestinal barrier, and the modulation of defense responses [4]. A great diversity of diseases, including cancer and inflammatory, autoimmune, metabolic, and neurological disorders, are associated with intestinal dysbiosis, a condition that results from imbalances in the gut microbiota composition and diversity [5,6]. Currently, therapeutic strategies aimed at restoring microbiota balance, such as the administration of prebiotics, probiotics, or probiotic-derived products, are receiving great interest [7].

Gut microbiota establishes a dynamic, complex, and bidirectional relationship with the intestinal epithelium and the immune system [8]. In the intestinal lumen, monitoring of microbes is mainly achieved by cells of the intestinal epithelium and the innate immune system through the expression of pattern recognition receptors (PRRs), which include toll-like receptors (TLRs), nucleotide-binding oligomerization domain (NOD)-like receptors, and retinoic acid-inducible gene (RIG)-like receptors [9]. These immune receptors interact with specific microbial-associated molecular patterns (MAMPs) and activate signaling pathways that trigger the expression of proinflammatory cytokines, chemokines, and antimicrobial peptides that help to control the gut microbial population [10,11]. Notably, the gut microbiota plays an essential role in priming the immune system. The immune system must learn to tolerate the commensal microbiota and respond appropriately to pathogens. In turn, the microbiota modulates the immune system to function properly [12]. Evidence indicates that germ-free mice exhibit significant immunologic deficiencies that could be reversed by inoculation with healthy murine microbiota [13]. Therefore, the gut microbiota allows activation of host immune responses that result in controlled basal inflammation, which assists in pathogen eradication and ensures tolerance to symbiotic microbiota and innocuous antigens [8,14].

Dendritic cells (DCs) in the lamina propria are essential to intestinal homeostasis due to their capacity to sample gut microbes and shape suitable immune responses [14]. In fact, DCs express tight junction proteins and can extend their dendrites between adjacent epithelial cells to directly detect luminal antigens or microorganisms [15]. These antigen-presenting cells are the gateway for foreign antigens and the initiation of immune responses. DCs integrate microbial signals and safeguard intestinal homeostasis by regulating the host immune system in response to gut microbes [16]. Activated DCs promote differentiation and activation of T-helper (Th) cells to specific CD4^+^ T-effector subsets through antigen-presenting molecules and cytokine secretion [17]. Activation of DCs by certain probiotic and beneficial bacteria leads to the secretion of transforming growth factor (TGF)-β and interleukin (IL)-10. These anti-inflammatory cytokines trigger differentiation of naïve T-cells to FoxP3^+^ regulatory T-cells (Tregs) that help to maintain immune tolerance to the gut microbiota [18,19]. Depending on the bacterial stimuli and secreted cytokines, DC activation can also drive Th effector responses (Th1, Th2, Th17/Th22) [20].

In addition to physical intercellular contact, DCs communicate with T-cells through released cytokines and extracellular vesicles (EVs) of endosomal origin called exosomes [21]. Exosomes are a heterogeneous group of lipid bilayer membrane vesicles that carry and deliver proteins, lipids and nucleic acids into recipient cells [22]. Their content can differ depending on the cell state (healthy or altered) and they regulate diverse cell processes, including the immune response and microbiota composition [23]. There is evidence that DC-derived exosomes can induce antigen-specific naïve CD4^+^ T-cell activation in vivo [24,25]. In addition to surface antigens and costimulatory molecules relevant for immune modulation, DC-derived exosomes contain microRNAs (miRNAs) [26]. The miRNAs are small non-coding RNAs (19–25 nucleotides) that post-transcriptionally regulate target messenger RNAs (mRNAs) by binding to complementary sequences at the 3′-untranslated region (3′-UTR), triggering mRNA degradation or protein synthesis inhibition. By this mechanism, miRNAs tightly regulate signaling pathways that control numerous cellular processes, and among them the immune response. Transport of miRNAs by secreted exosomes plays a pivotal role in modulating cross-talk between cells and represents a novel mechanism of intercellular communication that has proven critical in human health [26,27].

In the gut ecosystem, bidirectional microbiota–host communication does not depend on direct cellular contact. The intestinal epithelium is overlaid by a mucus layer that segregates the luminal microbes and host cells [28,29,30]. Therefore, microbiota–host communication is principally arbitrated by secreted bacterial factors that can penetrate the mucus layer and gain access to cells of the intestinal mucosa [31,32,33]. In addition to metabolites and soluble proteins, the microbiota releases bacterial extracellular vesicles (BEVs) [34,35]. Microbiota-derived BEVs are nano-sized membranous structures that act as a crucial mechanism for intra- and inter-kingdom communication, allowing long-distance delivery of bacterial effectors. Gut microbiota vesicles are equipped with numerous MAMPs and adhesins. They can cross the mucus layer and interact with IECs and DCs, activating PRR-signaling cascades that modulate defense and immune responses [36]. In addition to MAMPs, BEVs contain other bioactive molecules that influence host responses. In fact, some effects of BEVs are strain-specific as they depend on specific cargo produced by the parental strain.

Previous studies of our group provided evidence of the immunomodulatory and barrier protective effects of microbiota-derived vesicles, using intestinal *E. coli* strains as a model, specifically the probiotic *E. coli* Nissle 1917 (EcN) and commensal *E. coli* strains isolated from the stools of healthy human individuals [37,38]. We have shown that BEVs from these strains activate immune and defence responses in intestinal epithelial cells (IECs) [39,40,41,42], colonic explants [43] and an experimental mouse model of colitis [44]. Moreover, microbiota-derived BEVs modulate human DCs and the subsequent T-helper (Th) responses in a strain-specific manner [45]. The specific immunomodulatory effects of BEVs were mediated, at least in part, by miRNAs [46]. Specifically, EcN BEVs program DCs to drive the pro-inflammatory Th1 response needed for pathogen eradication, whereas ECOR12 BEVs modulate DCs towards a tolerogenic profile.

The aim of this work is to gain new insights into the mechanisms used by microbiota BEVs to orchestrate host immune regulation. It is known that in addition to direct cell-to-cell contacts, DCs communicate with neighboring T-cells through secreted mediators that include soluble released cytokines and EVs [47]. Here, we analyze the influence of microbiota vesicles on intercellular communication between DCs and T-cells by DC-secreted factors, using in vitro monocyte-derived DCs/naïve T-cells co-cultures. We further characterize the extracellular vesicles secreted by DCs in terms of costimulatory molecules and immune-related miRNA content.

## 2. Materials and Methods

### 2.1. Bacterial Strains and Isolation of Bacterial Extracellular Vesicles (BEVs)

The probiotic EcN (serotype O6:K5:H1) was provided by Ardeypharm (GmbH, Herdecke, Germany). ECOR12 is a commensal strain isolated from the fecal samples of a healthy human adult [38]. Strains were grown at 37 °C in Luria Bertani broth (LB) under constant rotation (150 rpm). BEVs were isolated from 1 L of LB culture grown in 2 L-Erlenmeyer flasks for 16 h, as previously described [45,48]. Cultures were centrifuged at 10,000× *g* for 30 min at 4 °C. The bacterial pellet was discarded and culture supernatants were filtered through a 0.22 μm pore filter (Merck Millipore, Danvers, MA, USA), concentrated by using Centricon Plus-70 centrifugal filters with a 100 kDa cut-off (Merck Millipore, MA, USA), and further filtrated to remove residual bacteria. BEVs were collected by ultracentrifugation at 150,000× *g* for 2 h at 4 °C, washed, and then resuspended in PBS (150 µL). Quantification of BEVs was performed by protein concentration using the BCA Pierce method. Microscopic image analysis of BEVs was previously assessed by Cryo-TEM [45]. Sterility was confirmed on LB plates, and aliquots were stored at −20 °C until use.

### 2.2. Generation and Stimulation of Human Monocyte Derived-DCs (mo-DCs)

Human mo-DCs were generated in vitro, as previously described [45]. Peripheral blood mononuclear cells (PBMCs) were isolated from fresh buffy coats of healthy donors by density gradient centrifugation using Histopaque 1077 (Sigma-Aldrich Chemical Co., San Luis, MO, USA). Monocytes were captured with magnetic-activated anti-human CD14 MicroBeads (Miltenyi Biotec, Bergisch Gladbach, Germany). To generate immature DCs (iDCs), monocytes were seeded at a density of 2 × 10^5^ cells/mL in 12-well plates or in 12 mm Transwell inserts, and cultured for 6 days (37 °C, 5%CO_2_) in mo-DC differentiation medium (Miltenyi Biotec), which included 800 IU/mL granulocyte-macrophage colony-stimulating factor (GM-CSF) and 1000 IU/mL IL-4. Fresh complete medium was added on the fourth day of culture. On day 6, immature mo-DCs (iDCs) were washed, kept in whole fresh mo-DC differentiation medium, and stimulated by the addition of BEVs (10 μg/mL) from EcN and ECOR12. This dose of BEVs did not cause cytotoxicity to DCs [45]. Unstimulated iDCs received the same volume of mo-DC medium and were incubated in parallel as a control.

### 2.3. DC/Naïve CD4^+^ T-Cell Co-Cultures

Naïve CD45RA^+^ CD4^+^ T-cells were isolated from human PBMCs by negative selection using the Human Naïve CD4^+^ T-Cell Isolation Kit II (Miltenyi Biotec, Bergisch Gladbach, Germany) and resuspended in fresh complete TexMACs™ medium (Miltenyi Biotec) at a density of 4 × 10^5^ cells/mL.

Following 6 h incubation with BEVs (10 µg/mL), mature mo-DCs were washed twice and co-cultured in TEXMACs medium with naïve T-cells in 12-well plates (direct co-culture) or in Transwell cell culture system (indirect co-culture) at a DC:T-cell ratio 1:2 by combining 2 × 10^5^ DCs (seeded in the apical inserts) with 4 × 10^5^ naïve T-cells (seeded in the lower chamber). On the fourth day of co-incubation, cell culture supernatants were filtered through a 0.22 μm pore filter and stored at −80 °C until use.

### 2.4. Cytokine Quantification

Secreted INF-γ, IL-4, IL-13, IL-17A, IL-22, and IL-10 were quantified in culture supernatants by using the human ProcartaPlex Multiplex Immunoassay (eBiosciences Inc., San Diego, CA, USA). Concentration of each analyte was detected using the MAGPIX instrument (Luminex Corp., Austin, TX, USA) in the facilities of the Scientific and Technological Centres of the University of Barcelona (CCiT-UB, Barcelona, Spain). Data were analyzed with xPONENT^®^ 4.2 software (Luminex Corp.). Secreted TGF-β was measured by an enzyme linked immunosorbent assay (ELISA) set (BD Biosciences, San Jose, CA, USA).

### 2.5. Isolati, on of DC-Derived Extracellular Vesicles (EVs)

Extracellular vesicles were isolated from mo-DCs cultures. For this approach, monocytes resuspended in mo-DC medium were seeded in T-25 flasks at a density of 1 × 10^7^ cells/mL and incubated for 6 days at 37 °C in 5% CO_2_. Then, iDCs were stimulated with BEVs from EcN and ECOR12 (10 μg/mL) for 16 h, washed and incubated in fresh Mo-DC differentiation medium for 72 h before isolation of DC-derived EVs. Unstimulated iDCs cells were processed in parallel as a control.

DC-derived EVs were isolated by differential ultracentrifugation as described elsewhere [49]. All the centrifugation steps were carried out at 4 °C. Briefly, 7 mL mo-DC cultures containing released EVs were centrifuged at 350× *g* for 15 min to pellet cells. Cell debris were removed from the supernatant by centrifugation at 2000× *g* for 30 min. Further centrifugation at 10,000× *g* for 45 min was applied to remove apoptotic bodies. The final supernatant was filtered using a 0.22 μm pore size filter and then subjected to ultracentrifugation at 100,000× *g* for 60 min. Pellets containing EVs were resuspended in PBS, and after a washing step, EVs were collected by ultracentrifugation at 100,000× *g* for 60 min. Pellets were resuspended in a final volume of 100 μL PBS, and protein concentration was measured using the BCA–Pierce method. Aliquots (20 μL) were stored at −20 °C until use.

### 2.6. Uptake of DiO-Labeled DC-EVs by T-Cells

DC-derived EVs (200 µg) were incubated with the fluorescent lipophilic probe Vybran*DIO (1% *v*/*v*) (Thermo Fisher Scientific, Waltham, MA, USA) for 1 h at 37 °C. Excessive Vybran*DiO was removed by ultracentrifugation at 100,000× *g* for 60 min at 4 °C. Labeled DC-EVs were resuspended in 20 μL PBS and stored at −20 °C until use. Naïve T-cells were seeded in an 8-well chamber slide at a density of 1.5 × 10^5^ cells/mL and grown for 24 h. DiO-labeled EVs (8 μg) were added to the culture media and incubated for 6 h. Then, lymphocytes were fixed with 4% PFA for 30 min. After PBS washing, nuclei were labeled with DAPI (1 mg/mL). Analysis of samples was carried out with a Leica TCS SP5 laser scanning confocal spectral microscope, using a 63× oil immersion objective lens. Images were captured with a Nikon color camera (16 bit). Fluorescence was recorded at 405 nm (blue, DAPI), 488 nm (green, Vybran*DIO). Images were analyzed using Fiji image processing package. Data were presented as mean ± standard error from three independent experiments. In all samples, the number of cells analyzed was between 60 and 100.

### 2.7. Cryo-Transmission Electron Microscopy (Cryo-TEM) of DC-Derived EVs

Cryo-TEM analysis was carried out with fresh DC-derived EVs samples resuspended in 0.1 M phosphate buffer (pH 7.2) in the Electronic Cryo-Microscopy Unit of the Scientific and Technological Centres of the University of Barcelona (CCiT-UB, Barcelona, Spain). Each EV suspension (5 μL) was placed on the carbon surface of a glow-discharged Lacey Carbon 300 mesh copper grid (Ted Pella, Redding, CA, USA). The sample was allowed to adsorb for 4 min at 100% humidity inside the chamber of a Vitrobot Mark III (FEI Company, Eindhoven, Netherlands). Excess liquid was blotted with filter paper, followed by cryo-immobilization by plunge freezing in liquefied ethane. The vitrified sample was stored in liquid nitrogen until its observation. Plunge-frozen samples were transferred to a Tecnai F20 microscope (FEI, Eindhoven, The Netherlands) using a cryo-holder system (Gatan, Pleasanton, CA, USA). The sample was examined at 200 kV, at a temperature ranging from −179 to −170 °C, using low-dose imaging conditions. Images were recorded with a 4096 × 4096-pixel CCD Eagle camera (FEI, Eindhoven, The Netherlands).

### 2.8. Nanoparticle Tracking Analysis (NTA)

Measurement of particle size distribution and concentration of DC-derived EVs by NTA was performed by the ICTS “NANBIOSIS” Unit of the Soft Materials/U6 at ICMAB-CSIC, using Nanosight NS300 (Particle Metrix, Germany) equipped with a with a 488 nm laser and a high sensitivity sCMOS camera. Samples were captured and analyzed at 25 °C following daily instrument calibration by applying the following settings: camera level 13, slider shutter 1232, slider gain 219, FPS 25, number of frames 1498, and 5 videos/10 s. Samples were diluted in PBS to an appropriate concentration before the analysis. Video acquisition was recorded in 11 positions for each sample with 5 cycles at each position and analyzed with the ZetaView analysis software.

### 2.9. Multiplex Surface Marker Analysis

Analysis of EV surface markers was performed using the bead-based multiplex MACSplex Exosome Human kit (Miltenyi Biotec), which detects 37 exosomal surface epitopes and two isotype controls. Briefly, samples of EVs were diluted with the MACSplex buffer to a concentration of 20 µg protein in a final volume of 120 µL. Then, 15 µL of antibody-coated MACSplex exosome capture beads were added to the samples and they were incubated overnight in constant rotation (450 rpm) at room temperature protected from light. The next day, the samples were washed once with MACSplex buffer and incubated with the MACSplex exosome Detection Reagents (APC-conjugated anti-CD9, anti-CD63 and anti-CD81 detection antibodies) for 1 h at room temperature protected from light in an orbital shaker (450 rpm), followed by three washes with MACSplex buffer. Then, 150 µL were transferred to flow cytometry tubes and analyzed in a Gallios flow cytometer (Beckman Coulter, Inc, Fullerton, CA, USA). A blank control composed by MACSplex buffer incubated with beads and detection antibodies was processed in parallel to measure the background signal, which was subtracted from the sample values. Median fluorescence intensity of all samples was normalized to the mean fluorescence intensity (MFI) of CD9, CD63 and CD81 markers for each sample. FlowJo Ver 10.2 (FlowJo LLC, Redding, CA, USA) was used to analyze cytometry data.

### 2.10. RNA Isolation

Total RNA from DC-derived EVs was isolated using the Total Exosome RNA & Protein Isolation kit (Invitrogen). External RNA was removed by RNase treatment and subsequent inactivation by freezing in liquid nitrogen. Exosome samples in 200 µL of PBS were mixed with 200 of 2X denaturing solution. After 3 min, 400 µL of acid-phenol-chloroform was added to each sample and centrifugated at 10,000× *g* for 12 min to obtain the aqueous phase. This sample was mixed with ethanol 100% and placed onto a spin column in a collection tube. After centrifugation at 10,000× *g* for 1 min, the column was washed successively with wash solution 1 and wash solution 2/3, and RNA was eluted with 10 µL of preheated nuclease-free water. RNA quality and concentration were assessed by the ratio of absorbance at 260 and 280 nm using a NanoDrop TM-2000 spectrophotometer (Thermo Fisher Scientific, Waltham, MA, USA).

### 2.11. miRNA Expression Analysis by RT-qPCR

For RT-qPCR, 5 ng/µL RNA was reverse transcribed using the miRCURY LNA. RT kit (Qiagen, Hilden, Germany) in a final volume of 10 µL. Quantification of selected miRNAs was performed in a StepOne Plus PCR cycler (Applied Biosystems, Foster City, CA, USA) using miRCURY LNA^TM^ miRNA PCR Assays (Qiagen) with gene-specific primers (Supplementary Table S1). RNU6-1 was used as endogenous reference gene for normalization. Expression stability across samples for RNU6-1 was assessed (Supplementary Figure S1). Relative gene expression was calculated as the fold change compared with the control by means of the 2^−ΔΔCt^ formula [50].

### 2.12. Ethics

Buffy coats from healthy donors were provided by the “Banc de Sang i Teixits” of Barcelona, according to the signed agreement with the Institution. The use of anonymous, non-identifiable human samples was approved by the Bioethics Committee of the University of Barcelona (Institutional Review Board: IRB00003099).

### 2.13. Statistical Analysis

Statistical analysis was performed using GraphPad Prism 7.0 software (GraphPad, San Diego, CA, USA) and data were expressed as mean ± standard error of the mean (SEM). Data are from at least three independent biological experiments. Significance analyses of the data were performed using one-way ANOVA followed by Tukey’s test. Significant differences were established at *p*-value ≤ 0.05.

## 3. Results

### 3.1. BEVs from Gut Microbiota Elicit a Distinctive Cytokine Profile in Human mo-DCs

**Figure 1:**
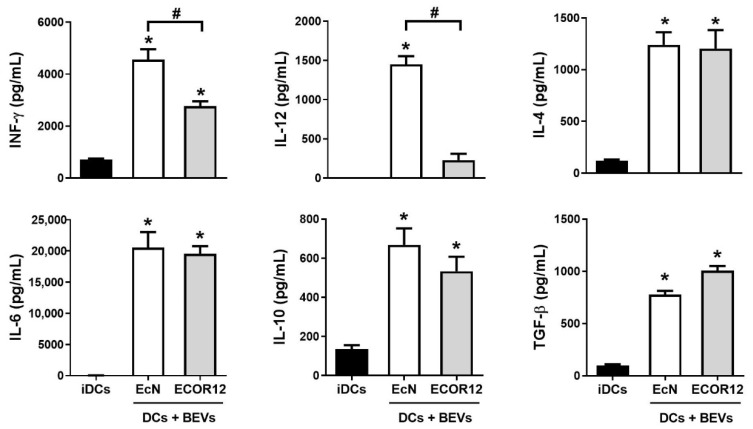
Activation of mo-DCs by BEVs from the probiotic EcN and the commensal ECOR12. Levels of INF-Υ, IL-12, IL-4, IL-6, IL-10, and TGF-β secreted by mo-DCs were quantified after 6 h incubation with BEVs (10 µg/mL) of the indicated strains. Untreated mo-DCs (iDCs) were used as a control (black bars). Data (mean ± SEM) are from three independent biological experiments (at least six donors) performed in triplicate. Statistical differences were assessed by one-way ANOVA followed by Tukey’s test (*p* < 0.05). * Significance versus untreated control DCs, # significance between DCs stimulated with EcN or ECOR12 BEVs.

It is well-known that BEVs transport components of bacterial cells and are relevant players in microbiota–host communication in the intestinal environment. In previous studies, we have shown that microbiota-derived vesicles have relevant functions for the priming of DCs and derived adaptive immune responses [45,46]. To further characterize the mechanisms involved, we here challenged human immature mo-DCs grown in Transwell inserts with BEVs (10 µg) isolated from two gut resident *E. coli* strains, the probiotic EcN and the commensal ECOR12. After 6 h of incubation, maturation of DCs was assessed by flow cytometry analysis using the Mo-DC differentiation inspector kit (Miltenyi Biotec) (Supplementary Figure S2) and cytokine production was quantified in culture supernatants by specific immunoassays. We focused on the main cytokines driving the T-helper responses, including INF-γ and IL-12 (drivers of Th1), IL-4 (driver of Th2), IL-6 (driver of Th17), TGF-β (driver of Treg) and the multifunctional IL-10 (Figure 1).

BEVs from both strains significantly induced secretion of all cytokines evaluated in comparison with non-treated DCs. However, significant differences in the Th1 and Treg polarizing cytokines were observed between DCs stimulated with EcN or ECOR12 vesicles. DCs stimulated with BEVs from probiotic EcN secreted higher levels of INF-γ and IL-12 than DCs stimulated with BEVs from the commensal ECOR12. In contrast, the highest level of the Treg-polarizing cytokine TGF-β was achieved by treatment with ECOR12 BEVs. BEVs from the probiotic EcN could also induce TGF-β, but to a lesser extent. BEVs from both EcN and ECOR12 activated secretion of Th2 and Th17 driver-cytokines (IL-4 and IL-6) and IL-10, without significant differences between strains (Figure 1).

### 3.2. Indirect and Direct Co-Cultures of BEV-Activated DCs/Naïve CD4^+^ T-Cells Generate Th-Specific Responses

**Figure 2:**
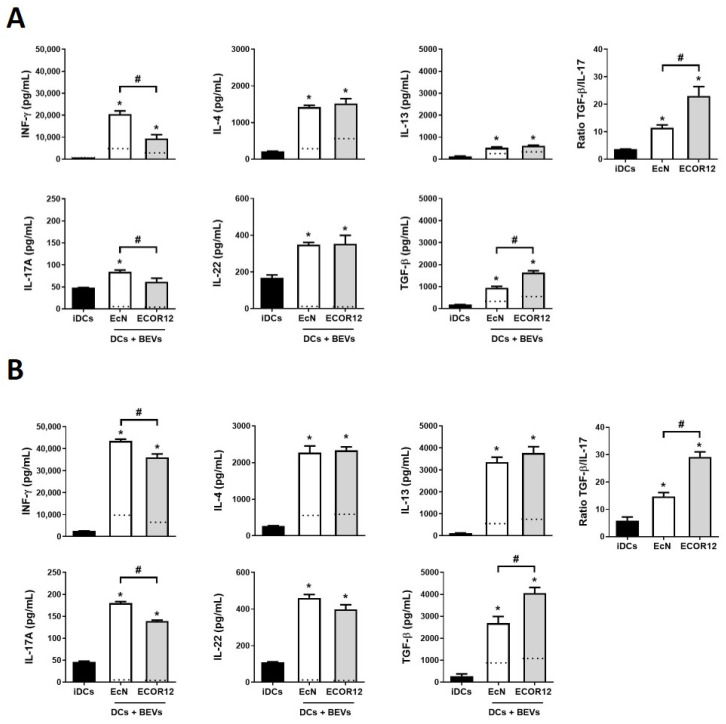
BEV-stimulated DCs activate naïve CD4^+^ T-cells in a strain-specific manner. Quantification of secreted INF-γ, IL-4, IL-13, IL-17, IL-22 and TGF-β by 4-day indirect (**A**) or direct (**B**) co-cultures of DCs stimulated with BEVs (10 µg/mL) from the intestinal *E. coli* strains EcN (white bars) or ECOR12 (grey bars) and naïve CD4^+^ T-cells at a DC:T-cell ratio of 1:2. Co-cultures with untreated iDCs were processed as a control (black bars). Indirect co-cultures were carried out in Transwell plates, where DCs (inserts) and naïve T-cells (basolateral chamber) were physically separated by a permeable membrane. In the direct model, co-cultures were carried out in 12 well-plates, allowing direct DC-T cells interaction. Dotted lines indicate the level of secreted cytokines by single activated DCs, incubated in the absence of T-cells. The ratios of TGF-β/IL-17 values are shown in the right panels (**A**,**B**). Data (mean ± SEM) are from three independent biological experiments performed in triplicate. Statistical differences were assessed by one-way ANOVA followed by Bonferroni’s test (*p* < 0.05). * Significance versus untreated control DCs, # significance between DCs stimulated with EcN or ECOR12 BEVs.

In previous studies, we reported the ability of mo-DCs stimulated by BEVs from intestinal *E. coli* strains to differentially regulate activation of allogenic CD4^+^ T-cells obtained from PBMCs of healthy donors towards specific effector subsets [45]. There is evidence that noncognate activated T-cells can modify the phenotypic and functional features of mature mo-DCs through costimulatory molecules. However, these effects cannot be mediated by non-activated T-cells [51]. To exclusively evaluate the immune responses activated by microbiota BEVs in the absence of crossed signals provided by T-cells activated by different stimuli in human donors, here we sought to analyze the immunomodulatory properties of BEVs from the probiotic EcN and the commensal ECOR12 in co-cultures of BEV-activated mo-DCs with naïve CD4^+^ T-cells. Two in vitro models of DC/naïve CD4^+^ T-cell co-cultures were set up. In the direct model, BEV-activated DCs were co-cultured with naïve CD4^+^ T-cells in 12-well plates. In this model, besides secreted polarizing cytokines and factors, activated DCs physically communicate with naïve CD4^+^ T-cells by direct intercellular contact through antigen presentation and surface costimulatory molecules. In the indirect model, mature/activated DCs were grown on permeable Transwell culture inserts and co-cultured with naïve CD4^+^ T-cells seeded in the lower chamber of the Transwell device. In this model, communication between activated DCs and T-cells only depends on secreted factors. In both models, immature DCs were challenged with BEVs from EcN or ECOR12 for 6 h, and then washed to remove non-internalized BEVs before adding naïve T-cells. As a control, CD4^+^ naïve T-cells were co-cultured with non-treated immature DCs. After 4 days, secreted levels of INF-γ (Th1 lineage), IL-4 and IL-13 (Th2 lineage), IL-17A (Th17 lineage), IL-22 (Th22 lineage) and TGF-β (Treg lineage) were quantified (Figure 2). Single cultures of BEV-activated DCs in TEXMACs medium were processed in parallel for comparison, and the corresponding values are indicated by the dotted lines in Figure 2.

In both co-culture models, DCs exposed to BEVs from gut beneficial bacteria activated naïve T-cells and triggered higher levels of all the cytokines that were analyzed than untreated DCs. Levels of cytokines secreted by BEV-stimulated DCs in the absence of lymphocytes were between two and four-fold lower than levels quantified in the corresponding co-culture models (Figure 2, dotted lines). These results supported the contribution of T-cells to cytokine production. This is especially relevant in the indirect DC/naïve T-cell co-culture model, where in the absence of close intercellular contact, communication with naïve T-cells depends exclusively on DC-secreted secreted factors.

The results showed that both direct and indirect co-cultures tend to induce similar T-effector responses. Except for IL-22, higher levels of secreted cytokines were achieved by direct DC/T cell co-culture. BEVs from both strains activated the Th2 response with similar levels of IL-4 and IL-13 between strains (Figure 2). In addition, no differences in the Th22 response were observed between DCs stimulated with EcN or ECOR12 BEVs. The main differences between BEV-mediated effects were in the Th1 and Treg responses. Although BEVs from both strains activated INF-γ and TGF-β production, probiotic EcN vesicles triggered a stronger Th1 response than ECOR12 BEVs. On the contrary, DCs stimulated with BEVs from the commensal ECOR12 triggered greater secretion of the unique Treg cytokine TGF-β than EcN BEVs. In addition, ECOR12 BEVs elicited lower secretion of IL-17A (Th17 response) than BEVs from probiotic EcN. The ratio TGF-β/IL-17 indicated that cell responses induced by ECOR12 BEVs were principally directed towards TGF-β secreting Treg cells, while probiotic EcN BEVs elicited more balanced Treg/Th17 responses (Figure 2).

### 3.3. Characterization of EV Secreted by mo-DCs in Response to Microbiota BEVs

**Figure 3:**
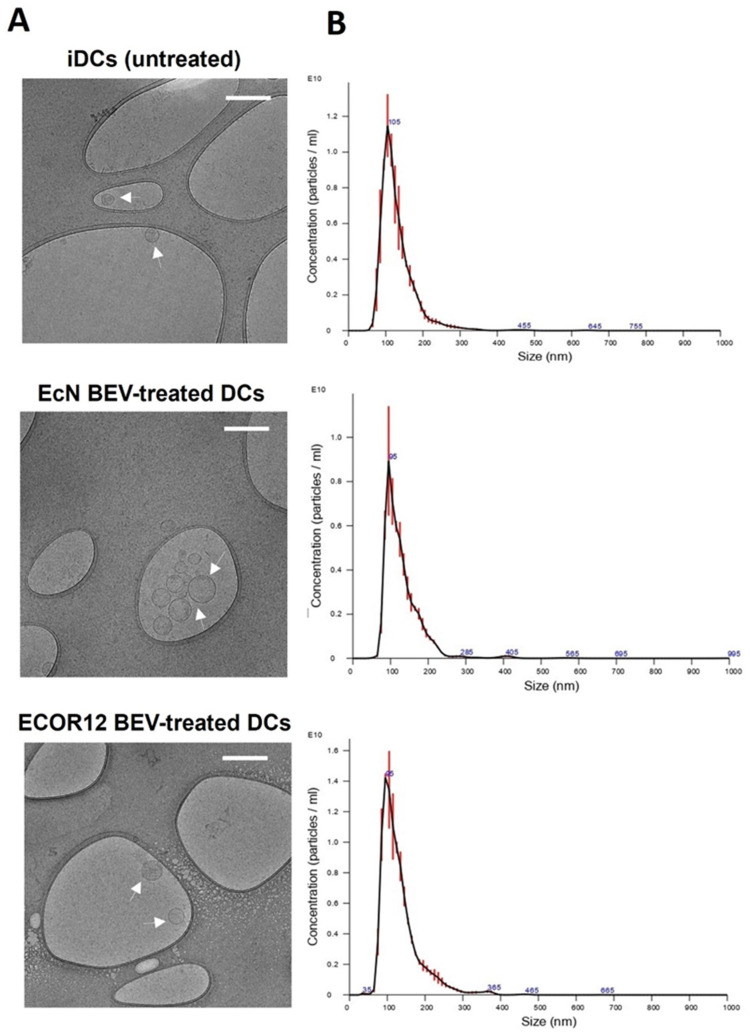
Characterization of EVs produced by mo-DCs in response to BEVs from the probiotic EcN or the commensal ECOR12. DC-derived EVs were isolated from culture supernatants by differential ultracentrifugation. Samples correspond to the final 100,000× *g* pellet. (**A**) Cryo-TEM observation of EV samples collected from iDCs (untreated) or DCs treated with vesicles from the indicated strains. Representative images of plunge-frozen EVs are shown. Scale bars: 200 nm. (**B**) Particle size distribution analyzed by nanoparticle tracking analysis and processed using NTA software (NanoSight). The data represent the mean ± SEM (error bars) of three independent analyses.

Results from indirect co-culture of mature DCs/naïve CD4^+^ T-cells indicate that, besides direct cell-to-cell contact, DCs communicate with neighboring T-cells through secreted mediators that include soluble released cytokines and extracellular vesicles. Knowing that vesicles from the probiotic EcN and the commensal ECOR12 elicited specific cytokine secretion profiles in mo-DCs (Figure 1) particularly differing in Th1 and Treg polarizing cytokines, we sought to isolate and characterize EVs secreted by DCs in response to vesicles from these gut microbiota strains. Human mo-DCs were stimulated with BEVs of EcN or ECOR12 (10 µg/mL) for 16 h. As a control, non-treated immature mo-DCs were processed in parallel. Then, the culture medium was removed, and cells were washed and cultured in fresh medium for 72 h to allow secretion of EVs. DC-derived EVs were isolated by differential ultracentrifugation. The size and structure of isolated EVs were analyzed by NTA and Cryo-TEM (Figure 3).

Results from three independent experiments showed no significant differences in the amount, size or structure of EVs produced by immature DCs or BEV-stimulated DCs. All EV samples exhibited a similar spherical shape with a single layer (Figure 3A). Regarding size distribution, all EVs showed a homogeneous narrow particle distribution with mean values of 128.8 ± 1.6 nm for untreated DCs, 130.26 ± 2 nm for EcN-EV-treated DCs and 134.06 ± 1.4 nm for ECOR12- EV-treated DCs (Figure 3B). This size distribution is compatible with small EVs in the size range of 50–150 nm. This finding is in accordance with the protocol used for EV isolation [49]. Likewise, the average number of particle counts was similar in all EV samples with no significant differences between untreated iDCs (7.15 × 10^10^ ± 3.29 × 10^9^ particles/mL) and BEV-treated DCs (6.4 × 10^10^ ± 4.28 × 10^9^ particles/mL for cells treated with EcN BEVs and 6.18 × 10^10^ ± 3.65 × 10^9^ particles/mL for cells treated with ECOR12 BEVs). Consistent with these data, total protein concentration was similar between the three samples. Concentration values measured by the BCA-Pierce method were 1630.96 ± 218.23 µg/mL for iDCs-derived EVs, 1518.35 ± 225.98 µg/mL for EVs isolated from culture supernatants of DCs stimulated with EcN BEVs and 1532.91 ± 293.97 µg/mL in the case of DCs stimulated with ECOR12 BEVs.

### 3.4. EVs Secreted by BEV-Activated DCs Express a Different Profile of Surface Antigens and Costimulatory Molecules

**Figure 4:**
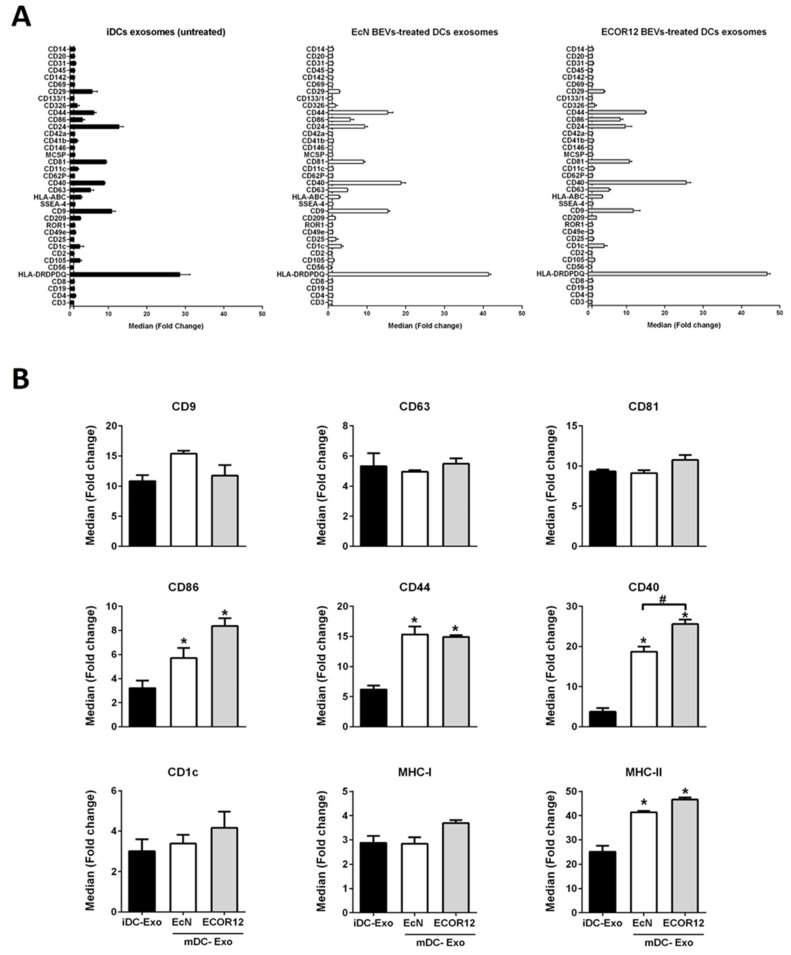
Extracellular vesicle surface marker values quantified by the bead-based multiplex MACSplex Exosome Human kit (**A**) and the main differences between the three subgroups (**B**): exosomes from untreated iDCs (black bars), exosomes from DCs treated with EcN vesicles (white bars) and exosomes from DCs treated with ECOR12 vesicles (grey bars). Statistical differences were assessed by one-way ANOVA followed by Tukey’s test (*p* < 0.05). * Significance versus untreated control DCs, # significance between DCs stimulated with EcN or ECOR12 BEVs.

The protein cargo of DC-derived EVs depends on the cell status and the stimuli received. Besides the several proteins that participate in vesicle biogenesis, DC-derived EVs contain surface markers and costimulatory molecules involved in cell-to-cell communication that greatly influence their immunological function, specially directing T-cell responses. Here, we analyzed the surface protein profile of DC-derived EVs by using the bead-based multiplex MACSplex Exosome Human kit (Miltenyi Biotec), which allows detection of 37 exosome surface epitopes (Figure 4A). EVs isolated from iDCs and BEV-treated DCs were incubated with capture beads following the manufacturer’s protocol. After incubation, bulk bead-captured EVs were subsequently detected by counterstaining with APC-labeled detection antibodies against the tetraspanins CD9, CD63 and CD81, which are often referred to as common exosome surface markers. All samples expressed these markers without significant differences between untreated and BEV-stimulated DCs (Figure 4B), suggesting that the main subpopulation of the isolated EV fractions corresponds to exosomes. Although the method used here to isolate DC-derived EVs does not exclude the presence of other type of small vesicles, we will refer to DC-derived EVs as exosomes to avoid confusion between extracellular vesicles released by bacteria and extracellular vesicles released by DCs.

Results showed that among the surface markers that were analyzed, CD86, CD44, CD40 and MHC-II exhibited greater expression in BEV-activated DCs than in untreated iDCs. Exosomes derived from DCs challenged with the commensal ECOR12 vesicles expressed higher levels of CD40 than exosomes isolated from EcN-treated DCs. The expression of CD86 follows the same trend, although the differences were not statistically significant (Figure 4B). Importantly, CD40, CD44 and CD86 are costimulatory molecules involved in activation, proliferation or differentiation of T-cells. Exosomes derived from DCs also transport markers related to microbial antigen presentation (Figure 4B). In addition to MCH-II, which is significantly enriched in BEV-activated DCs, MHC-I and CD1c were expressed in exosomes from untreated and stimulated DC. For these two markers, a tendency for increased expression was observed in exosomes from ECOR12-treated DCs, but the result was not significant.

**Figure 5:**
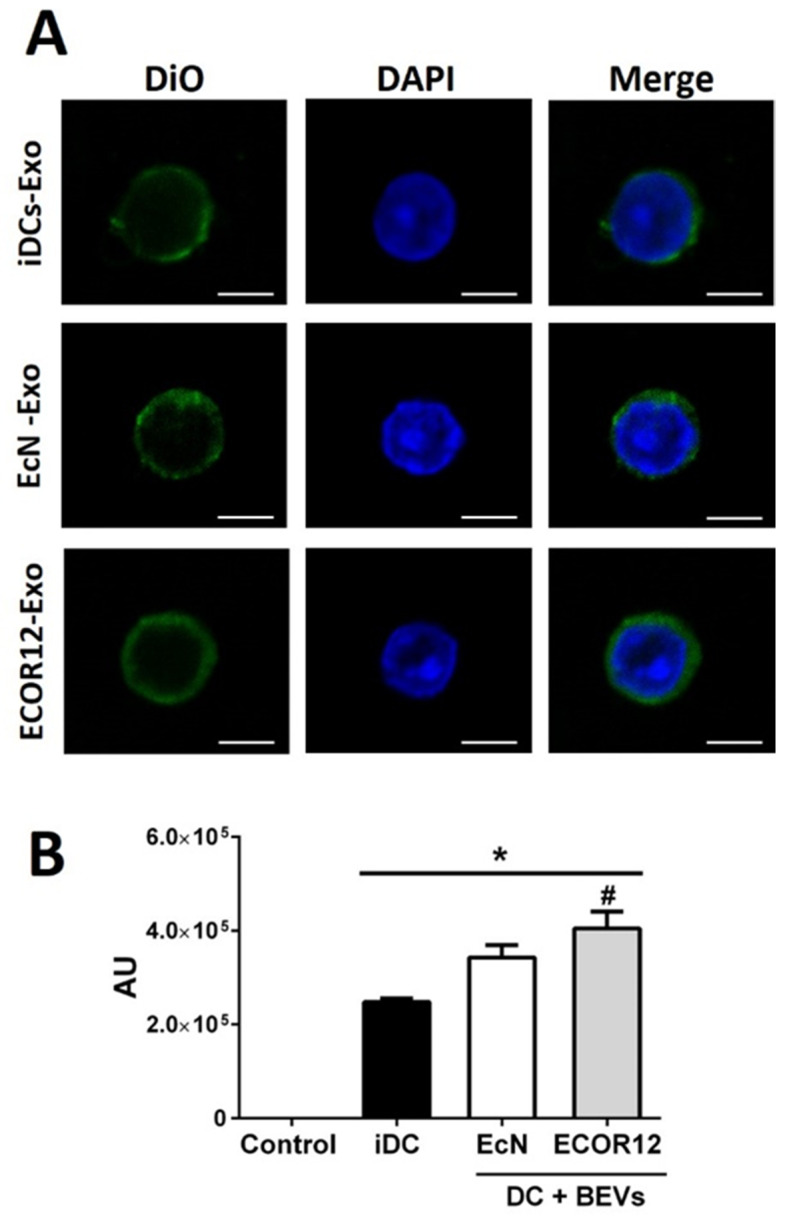
Exosomes derived from BEV-activated DCs are internalized by naïve CD4^+^ T-cells. (**A**) DIO-labeled DC-derived exosomes (green) isolated from untreated immature DCs or DCs stimulated with EcN or ECOR12 BEVs were cultured for 24 h with naïve CD4^+^ T-cells and analyzed by confocal microscopy. Nuclei were stained with DAPI (blue). Images were analyzed using Fiji image processing package. Scale bars: 5 µm. (**B**) T-cell-associated fluorescence (AU) was recorded for each exosomal sample. In all cases, the number of cells analyzed was between 60 and 100. Data were expressed as mean ± SEM from three independent experiments. * Significance versus exosomes isolated from untreated control iDCs, # significance between exosomes isolated from DCs stimulated with EcN or ECOR12 BEVs.

We next evaluated whether the isolated exosomes interacted with naïve T-cells. Freshly isolated naïve CD4 + T-cells were incubated for 24 h with DIO-labeled exosomes and analyzed by confocal microscopy. The results showed that exosomes from all DC samples were internalized by naïve T-cells. Significantly higher fluorescence intensity was achieved in T-cells challenged with exosomes derived from BEV-treated DCs than exosomes derived from iDC (Figure 5).

### 3.5. DC-Derived EVs Differentially Carry miRNAs Involved in Immune Responses

**Figure 6:**
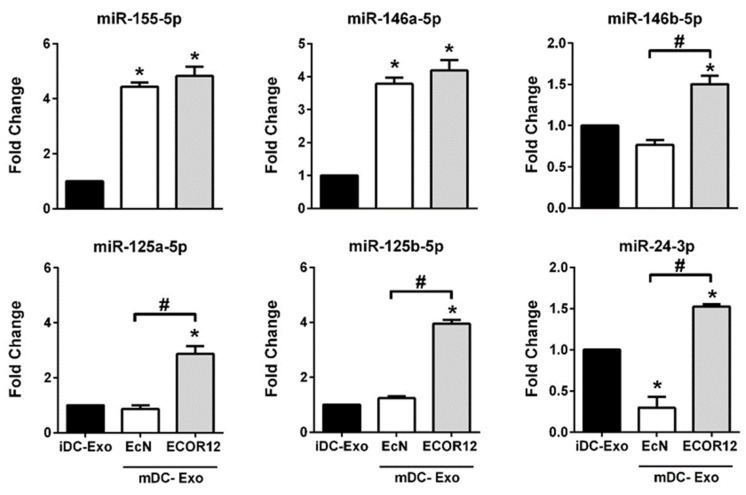
RT-qPCR expression analysis of selected miRNAs in exosomes isolated from DCs challenged with BEVs of the indicated *E. coli* strains. Immature DCs were stimulated for 16 h with BEVs (10 µg/mL) from EcN (white bars) or ECOR12 (grey bars). Cells were washed twice and further incubated for 72 h in fresh medium before isolation of exosomes by ultracentrifugation. Untreated immature DCs were processed in parallel (black bars). Relative miRNA levels of selected miRNAs known to be modulated by BEVs from EcN and ECOR12 in DCs were measured by RT-qPCR and normalized to the U6 reference gene. Data (mean ± SEM) are from three independent biological experiments (six donors) performed in triplicate and are presented as fold-change compared to untreated control cells. Statistical differences were assessed by one-way ANOVA, followed by Tukey’s test (*p* < 0.05). * Significance against untreated control cells; # significance between cells stimulated with EcN MVs or ECOR12 BEVs.

Among the cargo bioactive molecules of EVs, miRNAs have a key role as posttranscriptional regulators of a great variety of cellular and molecular processes. Previous studies by our group showed that BEVs isolated from probiotic and commensal *E. coli* strains elicit differential regulation of miRNAs in DCs. A set of regulated miRNAs correlated with the cytokine profile and derived specific T-cell responses driven by BEV-activated DCs [46]. Here, we sought to evaluate the presence of some of these immune-related miRNAs in DC-derived exosomes by RT-qPCR. First, we assessed miRNAs relative to DC activation, specifically miR-155-5p and miR-146a-5p (Figure 6). These are common miRNAs upregulated in DCs by BEVs from the probiotic EcN and the commensal ECOR12 [46]. Consistently, similar levels of miR-155-5p and miR-146a-5p were detected in exosomes isolated from BEV-stimulated DCs, without significant differences between strains. These results are consistent with their role in the activation and maturation of dendritic cells. The expression of miR-146b-5p, miR-125a-5p, miR-125b-5p and miR-24-3p, which were associated with IL-10 production and lower secretion of proinflammatory cytokines (INF-Υ, IL-12) in DCs treated with ECOR12 vesicles [46], were also quantified by RT-qPCR in the isolated exosomes (Figure 6).

Consistently, exosomes derived from DCs stimulated with BEVs from ECOR12 expressed higher levels of these miRNAs than exosomes derived from DCs stimulated with BEVs from probiotic EcN or untreated DCs. The expression level of miR-146b-5p, miR-125a-5p and miR-125b-5p in exosomes produced by EcN-stimulated DCs did not significantly differ from that detected in control exosomes released by immature DCs. By contrast, miR-24-39 was underrepresented in exosomes secreted by DCs in response to the probiotic EcN BEVs (Figure 6). The differential expression of these miRNAs between exosomes derived from DCs challenged with EcN or ECOR12 vesicles is consistent with their ability to differentially drive activation of naïve CD4^+^ T-cells towards the Treg (ECOR12 BEVs) or Th1 (EcN BEVs) effector responses.

## 4. Discussion

There is accumulating evidence on the essential role of the gut microbiota in the development and function of the host immune system. A healthy microbiome instructs the innate immune system to function properly through activation of balanced opposite pro- and anti-inflammatory responses that ensure a basal inflammatory state needed for pathogen eradication and, at the same time, safeguard tolerance to symbiotic microbiota [52,53]. Microbiota-secreted EVs are key players in microbiota–host communication. Contrary to luminal bacteria, BEVs can freely diffuse though the mucus layer and interact with innate immune cells of the intestinal mucosa. Activation and maturation of intestinal DCs is the first step to initiate the polarization of T-helper cells that will drive the subsequent adaptative immune responses. Previous studies of our group proved the ability of BEVs from probiotic and commensal *E. coli* strains to activate DCs and derived CD4^+^ T-cell responses in a strain-specific manner [45]. Differential modulation of immune-related miRNAs by microbiota BEVs may partially account for the specific effects [46].

To gain new insights into the mechanisms that mediate activation of T-cell responses by BEV-stimulated DCs, here we focused on DC-secreted factors. The experimental model consisted of indirect BEV-activated mo-DC/naïve CD4^+^ T cell co-cultures in Transwell permeable supports to prevent physical interaction between cells. Direct co-cultures were processed in parallel for comparison. Importantly, the pattern of secreted cytokines obtained here in direct mo-DC/naïve T-cell co-cultures was comparable to the immune responses activated by EcN and ECOR12 BEVs in co-cultures involving allogenic CD4^+^ T-cells derived from buffy coats of healthy donors [45]. Significantly, direct and indirect co-cultures tend to induce similar T-effector responses. However, except for IL-22, levels of secreted cytokines were lower in the absence of direct cell contacts. In the indirect model, the main differences between BEV-immunomodulatory effects also influenced Th1 and Treg responses. Probiotic EcN vesicles generated a stronger Th1 response assessed by INF-ϒ secreted levels, whereas ECOR12 preferentially triggered Treg responses with a higher anti-inflammatory TGF-β/IL-17 ratio.

The results from indirect co-cultures revealed communication between activated DCs and naïve CD4^+^ T-cells despite the absence of direct cellular contacts. Such intercellular communication should be mediated by DC-released immune mediators. In this context, the cytokine profile of DCs (Figure 1) challenged with vesicles from the probiotic EcN (increased levels of Th1 polarizing cytokines INF-γ and IL-12) or from the commensal ECOR12 (increased levels of TGF-β) is consistent with subsequent T-cell-derived responses. In addition to cytokines, DCs release EVs to communicate distantly with neighboring immune cells and influence T-cell fate. In this sense, the morphology and size of EVs derived from BEV-activated DCs were similar to that of EVs released by immature DCs. In all samples, the size was compatible with their classification as small EVs [22]. In addition, the amount of EVs produced did not differ between samples isolated from BEV-treated or untreated DCs. There is controversial information in the literature concerning production of DC-derived EVs. Some studies indicated that DCs constitutively release EVs and this secretion is increased after LPS stimulation to mature DCs [54]. In contrast, other reports showed decreased release of EVs by LPS-stimulated DCs compared to immature DCs [55]. Notably, the concentration of DC-derived EVs depends on the isolation protocol and the methodology used for quantification [56].

Understanding the molecular and cellular mechanisms of T-cell activation by DC-derived EVs is of great interest for their therapeutic potential [57]. DCs are professional antigen presenting cells that establish direct intercellular contacts with other immune cells through surface proteins and costimulatory molecules that are essential to trigger signaling events that lead to efficient T-helper cell differentiation. Several reports provide evidence that DC-derived exosomes can deliver these relevant surface molecules needed to activate T-cell responses, and that the outcome may depend on the presence of other immune cells. In this context, EVs derived from antigen-pulsed bone marrow DCs were shown to regulate adaptative immunity in vivo by triggering CD4^+^ and CD8^+^ T-cell responses. These effects required the presence of bystander DCs [24,58]. There is evidence that exosomes released by activated DCs mediate transfer of MHC complexes and costimulatory molecules not only to T-lymphocytes, but also between different populations of DCs. By this last mechanism, recipient DCs may then indirectly participate in naïve T-cell priming and amplification of immune responses [24]. The outcome of in vitro studies in which antigen presentation is only mediated by isolated DC-derived EVs depends on the lymphocyte type and whether it is naïve or primed [25]. There is evidence that EVs from immature DCs can directly induce an MHC class II-dependent activation and proliferation of human blood-derived allogenic CD4^+^ T-cells, but not naïve T-cells [49]. However, on contact with noncognate activated bystander T-cells, the exosomes released by LPS-stimulated DCs promote stronger peptide-specific T-cell responses than exosomes released in the presence of non-activated T-cells. These findings indicate that exosome composition can be further modified on noncognate interaction between DC and T-cells [51].

To deepen knowledge of the mechanisms that mediate regulation of adaptative immunity by microbiota, we sought to analyze whether microbiota vesicles could influence the functional properties and cargo of DC-derived EVs. The results revealed that activation of mo-DCs by microbiota BEVs leads to significant changes in exosomal costimulatory molecules and miRNA profiles compared to non-treated immature DCs. These differences are in accordance with the Th responses elicited by EcN or ECOR12 BEVs. Identification of CD9, CD63 and CD81 surface markers in isolated DC-derived EVs revealed that these samples consisting of small EVs were enriched in exosomes [59]. In addition to these exosome markers, EVs in all samples contained MHC-I/MHC-II complexes and costimulatory molecules that are needed for antigen presentation and T-cell activation [24]. Notably, the surface markers CD86, CD44, CD40 and MHC-II exhibited greater expression in BEV-activated DCs than in untreated iDCs. Overexpression of major histocompatibility complex MCH-II in exosomes released by BEV-activated DCs may mediate antigen presentation to CD4^+^ T-cells or neighboring DCs. Efficient exchange of functional peptide-MCH-II complexes by exosomes requires CD86 [24], which is also enriched in exosomes derived from BEV-activated DCs. Comparable MCH-I levels in all exosome samples suggest that microbiota vesicles do not influence cytotoxic CD8^+^ T-cell responses. Besides antigen presentation, proper activation and differentiation of naïve T-cells by DCs requires a second signal provided by costimulatory molecules such as CD86 and CD40 [60,61]. Notably, both proteins are enriched in exosomes derived from microbiota-activated DCs, together with the stimulatory factor CD44. This multifunctional protein is involved in several immune functions that include cell adhesion to extracellular matrix, cell-to-cell interactions and modulation of T-cell survival and proliferation [62]. Likewise, other studies have identified in DC-derived exosomes molecules involved in T-cell differentiation including CD40, DC-SIGN and CD80 [49]. The maturation state of DCs affects the ability of DC-derived exosomes to activate immune responses. In fact, exosome-derived MHC-antigen complexes can be presented more efficiently to T-cells when they are produced by mature DCs than those generated by immature DCs. This fact can be explained by increased levels of MHC-II, ICAM-, and costimulatory molecules on exosomes from mature cells that participate in the immune synapsis [55,63].

In addition to surface proteins, DC-derived exosomes carry regulatory miRNAs. The activation and subsequent clonal expansion of antigen-specific lymphocytes are crucial in the adaptative immune response [64]. In this context, miRNAs are emerging as key controllers of T-cell differentiation and function, as their expression is dynamically controlled by extracellular costimulatory signals and cytokines [65]. Antigen stimulation of naïve T-cells leads to huge changes in gene expression that are required for T-cells to proliferate, acquire functional competence and modify their migratory properties [65]. Delivery of miRNAs through released exosomes provides a mechanism for miRNA exchange between immune cells, such as DCs and T-cells, or even between DCs. In this context, miRNAs that target key immunoregulatory molecules have a relevant role in fine-tuning the immune responses [26].

We focused on immune-related miRNAs known to be regulated in DCs by BEVs produced by the probiotic EcN and the commensal ECOR12 [46]. Quantification of the selected miRNAs in exosomes derived from immature DCs and BEV-stimulated DCs showed good correlation with their differential expression in DCs. In this context, the common upregulated miR-155-5p and miR-146a-5p were enriched in exosomes generated by BEV-stimulated DCs, without significant differences between bacterial strains. These results are consistent with their role in activation and maturation of DCs and as key regulators of the immune system, modulating functions relative to TLR signaling and cytokine production [66,67]. Particularly, miRNA-155 plays a critical role in CD4^+^ T-cell function by targeting multiple genes involved in cell activation and differentiation processes [68]. Specifically, miR-155 was shown to downregulate cytokines produced by Th2 cells [69]. Moreover, miR-155 also promotes Th1 differentiation and INF-γ release through modulation of INF-γ signaling by directly targeting INF-γRα chain and by repressing SHIP1, a negative regulator of the PI3K signaling pathway [70,71]. miR-155 also positively regulates Th17 differentiation and IL-17A production by targeting SOCS1, the negative regulator of the activator transcription factor JAK/STAT [72]. Concerning miR146a, this miRNA acts as a feedback brake that inhibits NF-KB activation to ensure a dynamic balance between pro- and anti-inflammatory signals. In T-cells, miR-146a deficiency results in increased proliferation and expression of pro-inflammatory cytokines and activation markers [73]. In addition, miR-146a is highly expressed in Treg cells and controls IFN-γ mediated Th1 responses [74]. In fact, loss of miR-146a in Treg cells resulted in increased production of INF-γ by CD4*+* and CD8*+* T-cells, without affecting IL-4, IL-5, or IL-17 levels [74]. Concerning the miRNAs that are differentially regulated by vesicles from the probiotic EcN and the commensal ECOR12 in DCs, our results revealed that they are also differentially exported through exosomes, which suggests regulatory mechanisms of microRNA sorting into EVs [75]. In this context, the anti-inflammatory miR-146b-5p, miR-125a-5p, miR-125b-5p and miR-24-3p were overrepresented in exosomes derived from DCs stimulated with ECOR12 BEVs compared to exosomes derived from DCs stimulated with EcN BEVs or untreated DCs. These miRNAs directly target effector pro-inflammatory cytokines. Particularly, miRNA 24-3p and miR-125b target IFN-ϒ, whereas miR-125a inhibits IL-12 and TNF-α [46,73]. Consistently, ECOR12 BEVs triggered reduced secretion of these pro-inflammatory cytokines in direct and indirect DC/T cell co-cultures. In addition, overexpression of miR-24-3p attenuates antigen processing and secretion of Th1 polarizing cytokines [76]. In nasopharyngeal carcinoma, exosomal miR-24-3p has been correlated with impaired T-cell function with reduced capacity for Th1/Th17 differentiation and enhanced production of Treg cells [77].

There is evidence that each type of DC-derived EVs promotes different effects on CD4^+^ T lymphocytes. Small vesicles primarily induce secretion of INF-ϒ in T-cells, whereas large vesicles trigger secretion of Th2 cytokines such as IL-13 [49]. Consistent with their size, EVs derived from BEV-activated DCs were associated with low secretion of IL-13 and high secretion of INF-ϒ. However, EcN vesicles elicited a stronger Th1 response than ECOR12 vesicles. Specific modulation and export of anti-inflammatory miRNAs by ECOR12 vesicles may account, at least in part, for the tolerogenic and anti-inflammatory properties of this innocuous commensal. This fact reinforces the role of exosomes in cell-to-cell communication as a mechanism for the delivery of specific miRNAs that act in the recipient cells [26].

Currently, therapies targeted at modulating DC function are receiving great interest. Intestinal homeostasis and human health depend on well-balanced Th17/Treg and Th1/Th2 responses, and DCs play a fundamental role in regulating such responses. Imbalanced immune responses play an essential role in the onset and progression of certain diseases, such as inflammatory bowel disease (predominant imbalanced pro-inflammatory responses) or cancer (predominant tolerogenic DC responses). Thus, the modulation of intestinal immunity by DCs may provide a novel strategy to treat these disorders. In this context, ex vivo generation of tolerogenic DCs through exposure to suitable antigens (intestinal self-antigen, probiotics and postbiotics) is a promising approach [78]. Immunotherapy based on DC-derived exosomes is also envisaged as a promising approach for cancer and IBD treatment [23,57]. However, further scientific evidence is needed to improve efficacy before application to clinical practice. Based on the results of this work, the ability of BEVs from probiotics and commensal microbiota to induce tolerogenic DCs suggests the potential application of BEVs to re-induce tolerance and re-establish immune homeostasis in IBD patients.

## 5. Conclusions

This study provides new insights into the molecular mechanisms used by the gut microbiota to modulate immune responses and safeguard intestinal homeostasis. In this regard, we have shown that BEVs from probiotic and commensal *E. coli* strains activate DCs in a strain specific manner, triggering regulatory events to keep balanced anti-/pro-inflammatory responses. DCs in the lamina propria can contact the luminal environment by extending pseudopodia through the epithelial monolayer and integrate diverse incoming signals delivered by BEVs released by the gut microbiota. These bacterial effectors set up specific programmes that lead DCs to coordinate suitable T-cell responses through several mechanisms that include activation of TLR signaling pathways, regulation of miRNA expression and differential release of immune mediators through exosomes. To our knowledge, this is the first study showing the influence of microbiota BEVs on the immunomodulatory cargo of DC-derived EVs.

## Data Availability

Cryo-TEM image data on DC-derived exosomes are available at https://figshare.com/s/d7e47cacf7392bfd47a1.

## Supplementary Materials

The following are available online at https://www.mdpi.com/article/10.3390/nu14020344/s1, Figure S1: Expression stability across samples for RNU6-1, Figure S2: Maturation of DCs treated with BEVs from the indicated *E. coli* strains, Table S1: MiRCURY LNA primers (Qiagen) for RT-qPCR quantification of selected miRNAs.
