# Supplementary material for: Modulation of Dendritic Cells by Microbiota Extracellular Vesicles Influences the Cytokine Profile and Exosome Cargo
Source: Nutrients. 2022 Jan 14;14(2):344. doi: 10.3390/nu14020344 (PMC8778470; doi:10.3390/nu14020344)
Supplement: Supplementary file 1 [file nutrients-14-00344-s001.zip › Supplementary Figure S1.pdf]

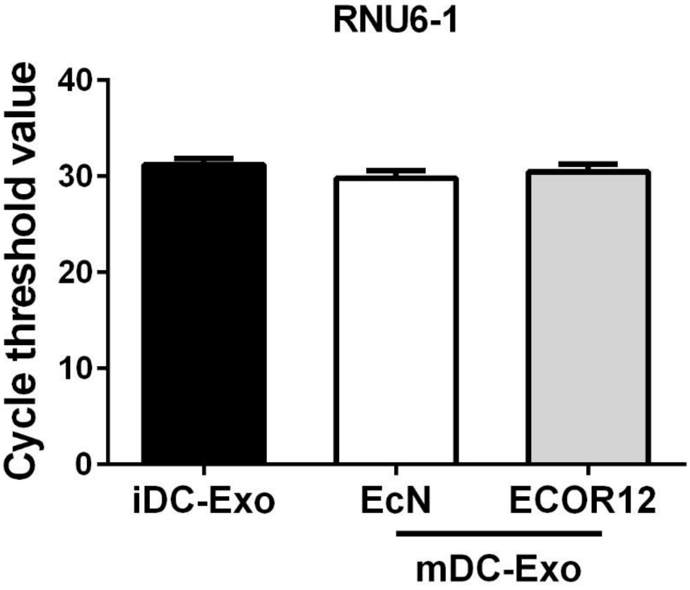

Supplementary Figure S1. Expression stability across samples for RNU6-1. RT-qPCR expression analysis of RNU6-1 in exosomes isolated from DCs challenged with BEVs of the indicated *E. coli* strains. Untreated immature DCs were processed in parallel.
