# Supplementary material for: Modulation of Dendritic Cells by Microbiota Extracellular Vesicles Influences the Cytokine Profile and Exosome Cargo
Source: Nutrients. 2022 Jan 14;14(2):344. doi: 10.3390/nu14020344 (PMC8778470; doi:10.3390/nu14020344)
Supplement: Supplementary file 1 [file nutrients-14-00344-s001.zip › Supplementary Figure S2.pdf]

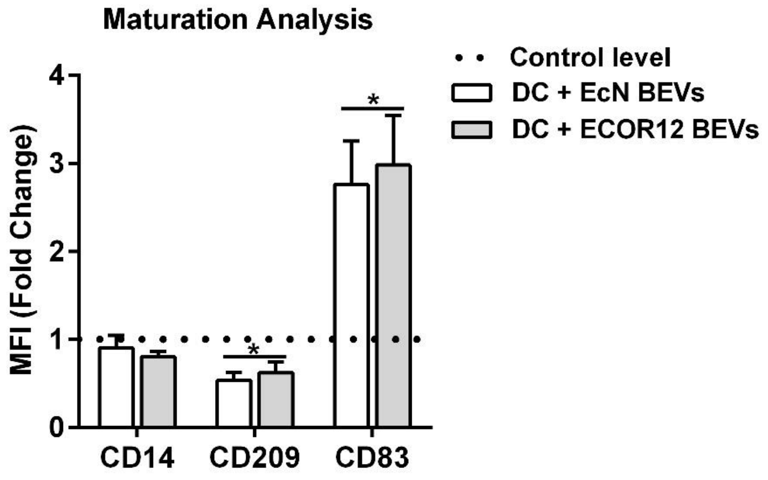

Supplementary Figure S2. Maturation of DCs treated with BEVs from the indicated *E. coli* strains. Quantification of CD14, CD83 and CD209 in mo-DCs exposed to BEVs (10 µg/ml) for 6 h was analysed by flow cytometry. Untreated mo-DCs were kept in DC medium as a control of immature DCs. The graph shows fold-changes in the mean fluorescence intensity (mean ± SEM) of the respective marker expression compared to control DCs (dot line). Data are from three independent biological experiments (at least three donors) performed in triplicate. Statistical differences were evaluated by one-way ANOVA followed by Tukey's test. *p <0.05 versus control DCs.
